# Why do family dementia caregivers reject caregiver support services? Analyzing types of rejection and associated health-impairments in a cluster-randomized controlled intervention trial

**DOI:** 10.1186/s12913-020-4970-8

**Published:** 2020-02-14

**Authors:** Ina Zwingmann, Adina Dreier-Wolfgramm, Alexander Esser, Diana Wucherer, Jochen René Thyrian, Tilly Eichler, Anika Kaczynski, Jessica Monsees, Armin Keller, Johannes Hertel, Ingo Kilimann, Stefan Teipel, Bernhard Michalowsky, Wolfgang Hoffmann

**Affiliations:** 1 European University of Applied Science (EUFH), Werftstrasse 5, 18057 Rostock, Germany; 2 University of Applied Science Hamburg, Hamburg, Germany; 3 German Alzheimer Association regional association Mecklenburg-Western Pomerania, Rostock, Germany; 4 German Center for Neurodegenerative Diseases (DZNE), Site Rostock/Greifswald, Rostock, Germany; 5 Institute of Medical Psychology and Medical Sociology, University Medicine Rostock, Rostock, Germany; 6 Department of Psychiatry and Psychotherapy, University Medicine Greifswald, Greifswald, Germany; 7 Department of Psychosomatic Medicine, University Medicine Rostock, Rostock, Germany; 8 Institute for Community Medicine, Department Epidemiology of Health Care and Community Health, University Medicine Greifswald, Greifswald, Germany

**Keywords:** Caregiver support, Caregiver supporting groups, Caregiver burden, Caregiver interventions, Randomized controlled trial

## Abstract

**Background:**

Although there are a number of support services accessible for most family dementia caregivers, many caregivers reject available and affordable support. Previous research suggests that rejections of support services may result from insufficient fit of available services with caregivers’ unmet needs and a lack of acknowledgement of caregivers’ unmet needs and associated support services. The present study investigates (a) the number, proportion and types of caregivers’ rejection on recommended tailored support, (b) socio-demographic and clinical determinants of caregiver’s rejection of both people with dementia (PwD) and caregivers, and (c) caregivers’ health-related variables related to caregivers’ rejection.

**Methods:**

Caregivers’ rejection of tailored support services was identified based on a standardized, computerized unmet needs assessment conducted by dementia-specific qualified nurses. The present analysis is based on data of *n* = 226 dyads of caregivers and their community-dwelling PwD who participated in a general practitioner (GP)-based, cluster-randomized intervention trial. The trial was approved by the Ethical Committee of the Chamber of Physicians of Mecklenburg-Western Pomerania, registry number BB 20/11. Data analyses were conducted using Stata/IC 13.1. We conducted Welch’s t-test, Pearson’s product-moment correlation, and conditional negative binomial regression models with random effects for GP to account for over-dispersed count data.

**Results:**

In sum, *n* = 505 unmet needs were identified and the same number of tailored recommendations were identified for *n* = 171 family dementia caregivers from the intervention group at baseline. For *n* = 55 family dementia caregivers not a single unmet need and recommendation were identified. A total of 17.6% (*n* = 89) of the recommendations were rejected by caregivers. Rejection rates of caregivers differed by type of recommendation. Whereas caregivers’ rejection rate on recommendations concerning mental health (3.6%), physical health (2.5%), and social, legal, and financial affairs (0%) were low, caregivers’ rejection rates concerning social integration (especially caregiver supporting groups) was high (71.7%). Thus, the rejections of family dementia caregivers are mainly linked to the delegation to caregiver supporting groups. Caregivers’ rejections were mainly related to personal factors of caregivers (*n* = 66), service-related factors (*n* = 6), relational factors (*n* = 1), and other factors (*n* = 17).

Furthermore, our results showed that the number of caregivers’ rejections was associated with a higher functional status of the PwD and are mainly associated with the rejection of caregiver supporting groups. Thus, caregivers visit supporting groups more often when the PwD shows low abilities in activities of daily living. Importantly, this is independent of the status of cognition and depression of the PwD as well as the physical and mental health of the family dementia caregivers.

**Conclusions:**

Our results underline the importance of understanding factors that determine caregivers’ rejection of support services. These need to be specifically addressed in tailored solutions for caregivers’ support services.

**Trial registration:**

ClinicalTrials.gov Identifier: NCT01401582 (date: July 25, 2011, prospective registered).

## Background

The large majority of people with dementia (PwD) wish to be cared for at home for as long as possible [1]. Given the fact that family dementia caregivers carry by far the largest burden of care for PwD, saving tremendous costs for national health care systems, supporting family dementia caregivers should be a major public health issue for the twenty-first century [2]. Family caregivers for PwD are the largest fraction among family caregivers. In comparison to other family caregivers, family dementia caregivers provide more hours and years of care and have poorer health outcomes [3]. Specifically, previous research revealed that caring for a PwD is particularly burdensome due to the irreversible and progressive nature of the disease, its long duration, and the deterioration in multiple areas of cognitive abilities, behavior, and personality [4]. Current research specified that the confrontation with cognitive impairment and behavioral symptoms (i.e., aggression and personality changes), and the need to assist in activities of daily living, are especially burdensome and distressing for caregivers [5, 6].

Previous research revealed that family dementia caregivers report high levels of burden as well as health impairments and consequently state a high number of perceived needs for more support and assistance [7, 8]. In general, there are two types of support services: (a) support services aimed directly at family caregivers (e.g., counselling services and caregiving courses, support groups for family caregivers, self help groups) and (b) support services primarily directed at those in need of care (i.e., PwD) (e.g., ambulatory care services, meals on wheels). In the present study, we focussed on support services aimed directly at family caregivers. Despite the reported high levels of perceived needs for more support and assistance of family dementia caregivers [7], previous research showed that family dementia caregivers often reject recommended support services and often only use them when they are no more able to psychologically or emotionally cope with the care situation [9]. Empirical studies confirmed that family dementia caregivers with high levels of health-impairments use significantly fewer caregiver support services compared to family dementia caregivers with low levels of health-impairments [10]. Analyzing of *n* = 5.923 family caregivers from six European countries, an empirical study by [1] revealed that only 3% of family caregivers use support services directly addressing their needs (i.e., support groups for family caregivers and internet-based information, self-help groups, caregiving courses, assistance services and home visits by social service providers). The results suggested that caregivers’ burden, gender, and education level all determine the use of support services. Thus, being male, higher educational level, and higher caregiver burden was associated with higher use of support services aiming directly on family caregivers [1]. As the number of community-dwelling PwD is rising, providing effective and tailored support for family dementia caregivers has important implications for providers as well as policy makers [11]. Although there is usually a variety of accessible support services for family dementia caregivers [12], many caregivers reject available and affordable support. However, the factors that influence decisions to use or reject support services for family dementia caregivers remain poorly understood [5].

Among the most widely used framework for analyzing decisions to use or reject support services for family dementia caregivers is the Andersen’s Health Behavioral Model (HBM [13];. The model comprises three factors that are crucial to predict and explain health services use. Predisposing factors are both social and individual characteristics that determinate a higher or lower propensity to use health services (i.e., demographics, social structure, and health beliefs). Social and financial enabling factors represent conditions that permit caregivers to satisfy their needs (i.e., community and personal enabling resources). Whereas predisposing and enabling factors are necessary but not sufficient conditions for caregivers’ service use, needs factors are sufficient conditions and must exist to use caregivers’ support services (i.e., evaluated needs and perceived needs).

**Fig. 1:**
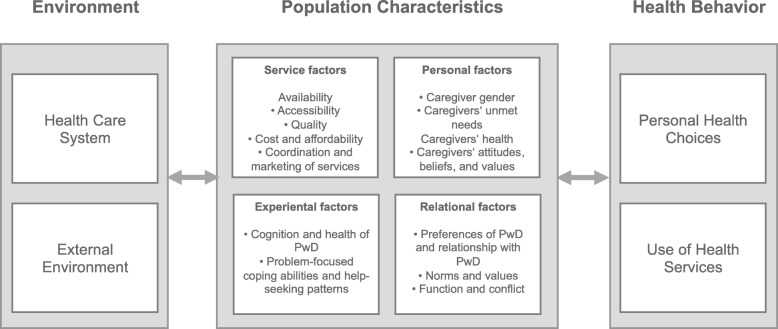
The adapted and extended Health Behavioral Model for family dementia caregivers in the present study (based on [13, 14])

Thus, Andersen’s HBM model emphasizes the importance of identifying and addressing unmet needs of family dementia caregivers to strengthen the use of support services [13]. A current review refined and extended Andersen’s HBM model and argued that decisions to use or reject support service for family dementia caregivers depend on four evidence-based dimensions: service factors, personal factors, experiential factors, and relational factors [14]. Service factors relate to service features that encourage or discourage its use (e.g., availability, accessibility, quality, cost), personal factors of family dementia caregivers impact their perception and actual use of support services (e.g., gender, unmet needs, health), experiential factors comprise challenges that affect there coping and decision making (e.g., caregiver burden, clinical characteristics of PwD), and relational factors reflect the relationship between caregiver and PwD (e.g., preferences of PwD, relationship with PwD). Figure 1 presents the adapted and extended HBM model for family dementia caregivers in the present study that is based on previous frameworks [13] [14];.

Previous research revealed that rejections of support services results mainly from a lack of acknowledgement of caregivers’ unmet needs and associated tailored support aimed at reducing the individual caregivers’ burden and health-impairments [15, 16]. Specifically, the identification of family dementia caregivers’ unmet needs was associated with better use of support services and contributed to the increased likelihood of PwD remaining in their homes (thereby reducing institutionalization) [15, 16]. Indeed, previous studies confirmed that family dementia caregivers show higher levels of unmet needs as well as lower levels of service utilization and lower identification rates of unmet needs by professionals compared to family caregivers for other chronic diseases [17]. A current review summarized that nurses who provide care management should assist family dementia caregivers to access supports and services that are tailored to their needs [14]. In summary, most authors have called for a comprehensive identification of caregivers’ unmet needs, an individualized tailored support strategy based on these unmet needs, and an accurate information and recommendation on support services for family dementia caregivers optimally provided by qualified nurses [14].

By following this call for research, the present study implemented a standardized, computerized unmet needs assessment of family dementia caregivers by dementia-specific qualified nurses. Based on this assessment of individual needs the nurses provided support to the family caregiver to access support and services that are tailored to their needs [6, 18]. Could this strategy lead to a zero rejection rate of family dementia caregivers on recommended tailored support services?

In the absence of comprehensive primary data concerning caregivers’ rejection on recommended tailored support from dementia-specific qualified nurses based on family dementia caregivers’ unmet needs, the objective of the present study is to investigate the number, proportion and types of caregivers’ rejection of these recommended tailored caregiver support services, and to identify associated socio-demographic and clinical characteristics of both PwD and caregivers. Finally, we assessed the impact on caregivers’ burden and health-related outcomes.

## Methods

### Trial design and participants

The present analyses are based on data of *n* = 226 dyads family dementia caregivers and their community-dwelling PwD recruited within a GP-based, cluster-randomized intervention trial [19, 20] (ClinicalTrials.gov Identifier: NCT01401582). Each of the participants received a comprehensive standardized, computer-based unmet needs assessment at the first time of measurement (i.e. baseline assessment). Based on the identified caregivers’ unmet needs, the computerized system generates an individual preliminary list of recommendations for specific caregivers’ support services. Furthermore, a dementia-specific qualified study nurses designed a tailored intervention plan for support services to which the family dementia caregivers could agree or reject. They were given time to consider how to respond and had the opportunity to get support from dementia-specific qualified study nurses at patients’ homes during a 6-months interval (i.e. intervention “dementia care management”). Enrolment into the main study began January 1, 2012 and finished December 31, 2014. The design, study procedures and instruments, and results of the trial have been explained elsewhere [20]. The intervention trial evaluated a collaborative Dementia Care Management (DCM) program that aims to provide optimum care to community-dwelling PwD and their family dementia caregivers (two arms: intervention versus control “care as usual” group, 1:1 simple randomization). From a total of *n* = 854 GPs in five municipalities of Mecklenburg-Western Pomerania, 16% (*n* = 136 GPs) gave written informed consent to take part in this trial. By using a screening instrument for cognitive impairment in dementia (DemTect, [21], the participating GPs screened patients for dementia (eligibility criteria: age ≥ 70 years, living at home, DemTect score < 9). This instrument is an interview-based screening containing five tasks (i.e., number transcoding task, delayed recall of word list, word fluency task, recall of word list, and digit span reverse) [22]. A total of *n* = 407 people screened positive for dementia gave written informed consent to participate and of these, *n* = 317 people screened positive for dementia provided the contact to a family caregiver (*n* = 226 family caregivers of the intervention group, *n* = 91 family caregivers of the control group). Since the assessment of unmet needs was conducted only in the intervention group, the sample under investigation in the present study is *n* = 226 family dementia caregivers and their community-dwelling PwD.

### Procedures and measures

Dementia-specific qualified study nurses performed a computer-based comprehensive unmet needs assessment at the participant’s home. The qualification of the dementia-specific qualified study nurses comprised seven modules (i.e., dementia health care supply and network, basics of health care supply, nursing, medical aspects, communication and counselling techniques, needs assessment, and practice period) [23]. The assessment of caregivers’ unmet needs included a battery of standardized questionnaires and tests (e.g., HABC-Monitor [24]). Depending on the caregiver’s answers and results, respectively, the system identified a preliminary list of unmet needs. Additionally, the dementia-specific qualified study nurses could add additional unmet needs that they had identified. The needs assessment was developed by German guidelines on dementia, meetings with experts and scientific advisory board, and reviews of current literature. Thus, it integrates a range of caregiving role domains and health-related outcomes (i.e., social, legal and financial affairs, family role conflicts, mental and physical health problems). A detailed description of the needs’ assessment and recommended caregivers’ support services of the present study is shown in Table 1. For a detailed explanation on the computer-based needs’ assessment, see [25]. Specifically, the system selects from a total of 19 modules of caregivers’ support services (four modules focusing on social integration, ten modules concentrating to mental health, four modules directed on physical health, and one module aiming at social, legal, and financial affairs).

**Table 1:** Types of recommendations based on caregivers’ unmet needs and potential evidence-based types of caregivers’ rejection

			Evidence-based types of caregivers’ rejection			
Types of recommendations based on caregivers’ unmet needs		**Measurements**	**Service factors**	**Personal factors**	**Experiential factors**	**Relational factors**
Social Integration	Delegation to caregiver supporting groups	BIZA-D (Zank et al., 2006)	X	X	X	X
	Consultation on personal constraints and challenges of caregiver	BIZA-D (Zank et al., 2006)		X	X	X
	Consultation on professional role conflicts of caregiver	BIZA-D (Zank et al., 2006)		X	X	X
	Consultation on family role conflicts of caregiver	BIZA-D (Zank et al., 2006)		X	X	X
Mental health	Consultation on depression and anxiety of PwD	HABC-Monitor (Monahan et al., 2012)		X	X	X
	Consultation on aggression and resistance of PwD	HABC-Monitor (Monahan et al., 2012)		X	X	X
	Consultation on hallucination and delusion of PwD	HABC-Monitor (Monahan et al., 2012)		X	X	X
	Consultation on sleep disturbance of PwD	HABC-Monitor (Monahan et al., 2012)		X	X	X
	Consultation on repetitive behavior of PwD	HABC-Monitor (Monahan et al., 2012)		X	X	X
	Consultation on impulsive behavior of PwD	HABC-Monitor (Monahan et al., 2012)		X	X	X
	Consultation on Safety of PwD	HABC-Monitor (Monahan et al., 2012)		X	X	X
	Consultation on behavior change of PwD	BIZA-D (Zank et al., 2006)		X	X	X
	Consultation on changes in personality and relationship between PwD and caregiver	BIZA-D (Zank et al., 2006)		X	X	X
	Consultation on quality of live and mental health of caregiver	HABC-Monitor (Monahan et al., 2012)		X	X	X
Physical health	Consultation on mobility, balance and falls of PwD	HABC-Monitor (Monahan et al., 2012)		X	X	X
	Consultation on physical health of caregiver	HABC-Monitor (Monahan et al., 2012)		X	X	X
Social, legal, and financial affairs	Consultation on social, legal, and financial issues of caregiver	HABC-Monitor (Monahan et al., 2012) BIZA-D (Zank et al., 2006)		X	X	X

Each unmet need contains of defined algorithms that comprise the trigger condition derived from standardized baseline that initiated recommendations of support services as well as criteria to control the task completion in subsequent home visits. Each recommendation list of support services was confirmed in the weekly case conference with an interdisciplinary expert panel (including neurologists, psychiatrists, psychologists, nursing scientists, and pharmacists). Accordingly, they confirmed or improved the recommendation list of support services [25]. Our previous study found an increase of 85% regarding recommendations of support services after the implementation of the computer-based needs assessment [25] and thus, underlying the complexity of their home caring situations. By assessing the practicability and acceptability of the computer-based needs assessment, the dementia-specific qualified study nurses (*n* = 6) evaluate the assessment as very helpful [25].

For the present analysis, the variables under investigation concerning caregivers were: number and types of rejections of recommended caregivers’ support services, relation to the PwD, education, age, sex, physical and mental health (12-Item Short Form Survey [26], hours spent for caregiving, employment status, income per month, as well as syndromes of somatization, depression, and anxiety (Brief Symptom Inventory [27]. Specifically, caregivers’ physical and mental health was assessed by a validated, economic instrument to assess multiple health dimensions and reduce respondent burden (SF-12 [26]). Caregivers’ syndromes of somatization, depression, and anxiety were assessed by a widely used and validated instrument that assesses the syndromes of somatization, depression and anxiety (BSI-18 [27]). With regard to PwD, we analyzed sex, age, functional status (Bayer Activities of Daily Living Scale, B-ADL [28]), living situation (alone/not alone), living in a partnership, depression (Geriatric Depression Scale, GDS, [29]) and cognitive status (Mini-Mental-Status-Test, MMSE, [30]). These variables were proofed to be linked with family dementia caregivers’ rejection and health impairments [for reviews see [11, 31] and comply with our study protocol [20].

### Statistical analyses

The statistical analyses and reporting follow our statistical analyses plan, CONSORT-statement and its extensions concerning cluster-randomized, pragmatic trials with non-pharmacological treatments [20] by using Stata/IC 13.1. Thus, we imputed missing data by multiple imputations via chained equations. While data were collected by study nurses in a personal interview at patients’ home, the rate of missing data ranged from 0% (e.g., caregivers’ gender, caregivers’ age) to 3.98% (depression of PwD). We controlled for random effects of GP’s to account for the stochastic dependency of PwD and caregivers treated by the same GP as well as predefined covariates in the present study. We analyzed the number, proportion, and types of caregivers’ rejection (see Table 2).

**Table 2:** Number, proportion, and types of caregivers’ rejection (*n* = 226)

Types of recommendations based on caregivers’ unmet needs	No. of recommendations	No. of caregivers’ agreement	No. of caregivers’ rejections	Proportion of caregivers’ rejection	Types of caregivers’ rejections						
					Relational factors	Personal factors of caregivers				Service factors	Other
					Undesired from PwD	Undesired	Unneccessary	No time	Med. treatment	Unavailable service	
Total	**505**	**416**	**89**	**17.6**	**1**	**43**	**10**	**12**	**1**	**6**	**17**
Social Integration	**106**	**30**	**76**	**71.7**	**1**	**39**	**10**	**10**	**1**	**6**	**9**
Delegation to caregiver supporting groups	96	21	75	78.0	1	38	10	10	1	6	9
Consultation on personal constraints and challenges of caregiver	2	2	0	0							
Consultation on professional role conflicts of caregiver	4	4	0	0							
Consultation on family role conflicts of caregiver	4	3	1	25.0		1					
Mental health	**308**	**297**	**11**	**3.6**		**1**					
Consultation on depression and anxiety of PwD	39	38	1	2.6		1					
Consultation on aggression and resistance of PwD	18	18	0	0							
Consultation on hallucination and delusion of PwD	14	14	0	0							
Consultation on sleep disturbance of PwD	10	9	1	10.0		1					
Consultation on repetitive behavior of PwD	15	15	0	0							
Consultation on impulsive behavior of PwD	28	26	2	7.1				1			1
Consultation on safety of PwD	69	66	3	4.3							3
Consultation on behavior change of PwD	17	17	0	0							
Consultation on changes in personality and relationship between PwD and caregiver	13	13	0	0							
Consultation on quality of live and mental health of caregiver	85	81	4	4.7		1		1			2
Physical health	**79**	**77**	**2**	**2.5**							**2**
Consultation on mobility, balance and falls of PwD	67	65	2	3.0							2
Consultation on physical health of caregiver	12	12	0	0							
Social, legal, and financial affairs	**12**	**12**	**0**	**0**							
Consultation on social, legal, and financial issues of caregiver	12	12	0	0							

Nominal variables were presented by proportion and metric variables were summarized by means as well as standard deviations (*SD*) (see Tables 2 and 3).

**Table 3:** Characteristics of caregivers and their PwD (*n* = 226)

Characteristic	Sample (n = 226)	Bivariate relationship to caregivers’ rejection
Caregiver		
Gender		*t* = −0.361, *p* = 0.114
Female, %	73.0	
Male, %	27.0	
Age, mean (SD)	64.55 (12.87)	*r* = 0.003, *p* = 0.724
Currently Working, %	27.4	*t* = 0.182, *p* = 0.429
Relationship with PwD, %		
Spouse, life partner, Siblings	46.9	*t* = 12.997, *p* = 0.980
Son/daughter, Son-in-law/daughter-in-law, Grandchildren	49.6	*t* = 0.229, *p* = 0.321
Other	3.5	*t* = − 0.423, *p* = 0.568
Education, %		
Without degree	2.7	*t* = 15.432, *p* = 0.977
Lower Secondary Education	37.3	*t* = − 0.217, *p* = 0.657
Higher Secondary Education	31.1	*t* = − 0.651, *p* = 0.105
Polytechnical Degree	16.9	*t* = 0.094, *p* = 0.841
Advanced technical college certificate	2.2	*t* = 0.355, *p* = 0.457
Higher education entrance qualification	9.8	*t* = 0.285, *p* = 0.530
Income (net) per month, € (SD)	1828.29 (740.29)	*r* = − 0.001, *p* = 0.546
Hours spent for caring per month	141.29 (224.21)	*r* = 0.001, *p* = 0.183
SF-12 physical health of caregivers, mean (SD)	47.43 (9.24)	*r* = − 0.015, *p* = 0.176
SF-12 mental health of caregivers, mean (SD)	52.80 (9.11)	*r* = − 0.012, *p* = 0.269
BSI-18 somatization of caregivers	1.41 (2.25)	*r* = 0.046, *p* = 0.224
BSI-18 depression of caregivers	1.04 (2.56)	*r* = 0.045, *p* = 0.176
BSI-18 anxiety of caregivers	1.50 (2.63)	*r* = 0.057, *p* = 0.085
Person with Dementia (PwD)		
Gender		*t* = 0.315, *p* = 0.175
Female, %	61.6	
Male, %	38.4	
Age, mean (SD)	80.88 (5.56)	*r* = 0.029, *p* = 0.137
Living in partnership, %	54.0	
Living situation (living alone), %	47.8	*t* = 0.082, *p* = 0.708
Severity of dementia (MMST), mean (SD)	21.4 (5.45)	*r* = −0.008, *p* = 0.704
Depression (GDS)		*t* = 0.047, *p* = 0.876
Mild, %	84.8	
Moderate or severe, %	15.2	
Functional status (B-ADL), mean (SD)	4.32 (2.70)	***r*** **= 0.092,** ***p*** **= 0.021**

Footnote: *r* = Pearson’s product-moment-correlation coefficient, *t* = Welch’s t-test coefficient (two-sided), *p* = *p*-values, MMST = Mini Mental State Test ranging from 0 to 30 (higher score indicates better cognitive functioning), B-ADL = Bayer Activities of Daily Living Scale ranging 0–10 (lower score indicates better performance), GDS = Geriatric Depression Scale ranging from 0 to 15 (score ≥ 6 indicates depression), SF-12 = 12-Item short form survey assessing physical and mental health, BSI-18 = brief symptom inventory short form assessing syndromes of somatization, depression, and anxiety

We conducted Welch’s t-test and Pearson’s product-moment correlation to analyze the bivariate associations of socio-demographic and clinical characteristics with the number of rejections (see Table 3). For multivariate analyses we fitted conditional negative binomial regression models with random effects for GP to account for over-dispersed count data (see Table 4). We conducted the over-dispersion test by using Stata/IC 13.1. On average, caregivers had 2.19 unmet needs (SD = 2.15). 53.1% caregivers had one up to three unmet needs (*n* = 120), 18.6% (*n* = 42) had four up to six unmet needs, and 4.0% (*n* = 9) had seven or more unmet needs. Furthermore, the variance of the dependent variable number of caregivers` rejection (*var* = 22.84) is larger than the mean. Furthermore, the data is strongly skewed to the right (*skewness* = 1.85, *kurtosis* = 6.32) and thus, ordinary least squares regression analysis would be inappropriate. While showing greater variance than might be expected in a poisson distribution, the distribution of number of caregivers` rejection is displaying signs of overdispersion. Thus, we examined over-dispersion parameter alpha by conducting the likelihood ratio test. While the over-dispersion parameter alpha (*chibar* = 5.46, *p* = .01) is significantly different from zero, over-dispersed and is not sufficiently described by the simpler poisson distribution, we computed negative binomial regression models.

**Table 4:** Health-related factors associated with the number of caregivers’ rejections (*n* = 226)

	*b*	*z*	*p*	*CI*_*95−*_	*CI*_*95+*_
Covariates					
Caregiver gender (female)	−0.156	− 0.52	0.602	− 0.741	0.430
Caregiver currently working	0.440	1.39	0.165	−0.182	1.061
Caregiver age	0.008	0.62	0.535	−0.170	0.033
PwD gender	0.201	0.64	0.520	−0.413	0.816
PwD age	0.013	0.57	0.571	−0.032	0.059
PwD living situation (living alone)	−0.024	−0.09	0.928	−0.539	0.491
PwD severity of dementia (MMST)	0.024	0.94	0.346	−0.026	0.074
PwD depression (GDS)	−0.211	−0.67	0.506	−0.831	0.409
PwD functional status (B-ADL)	**0.136**	**2.53**	**0.011**	**0.031**	**0.241**
Predictors					
Caregivers SF-12 physical health	−0.022	−1.61	0.107	−0.048	0.005
Caregivers SF-12 mental health	−0.006	−0.38	0.707	−0.039	0.026
Caregivers SF BSI-18 somatization	−0.038	−0.67	0.500	−0.149	0.072
Caregivers SF BSI-18 depression	−0.013	−0.21	0.830	−0.128	0.102
Caregivers SF BSI-18 anxiety	0.055	1.00	0.319	−0.053	0.162
R ^2^					0.09

*Footnote:* Conditional negative binomial regression model with random effects for GP; Number of caregivers’ rejection was the predictor of interest; p-values are given one-sided, CI = Confidence interval, MMST = Mini Mental State Test ranging from 0 to 30 (higher score indicates better cognitive functioning), B-ADL = Bayer Activities of Daily Living Scale ranging from 0 to 10 (lower score indicates better performance), GDS = Geriatric Depression Scale ranging 0–15 (score ≥ 6 indicates depression), SF-12 = 12-Item short form survey assessing physical and mental health, BSI-18 = brief symptom inventory short form assessing syndromes of somatization, depression, and anxiety

In these models, we included caregivers’ health impairments (i.e., caregivers’ physical and emotional health, as well as syndromes of somatization, depression, and anxiety of caregivers), while adjusting for both caregivers’ variables (i.e., sex, employment status, age) as well as for variables of the PwD (i.e., sex, age, functional and cognitive status, depression, living situation).

## Results

The majority of caregivers were women (73.0%) with lower (37.3%) or higher (31.1%) secondary education, with an average of 141.3 h spent for caring per month, and with a mean age of 64.6 years. They mostly cared for female PwD (61.6%) with an average age of 80.9 years, showing moderately impaired functional (*B-ADL*_*mean*_ = 4.32) and cognitive status (*MMST*_*mean*_ = 21.4). In sum, *n* = 505 tailored recommendations of support services across 17 different categories were identified for *n* = 171 caregivers. Specifically, 75.7% caregivers received at least one recommendation (*n* = 171), whereas only 24.3% caregivers obtained no recommendation (*n* = 55). Thus, for solely *n* = 55 family dementia caregivers not a single recommendation were identified. The number of recommendations ranged from none (minimum) to twelve (maximum) with an average of 2.19 (*SD* = 2.15). 53.1% caregivers received one up to three (*n* = 120), 18.6% (*n* = 42) obtained four up to six, and 4.0% (*n* = 9) get seven or more recommendation. A total of 17.6% (*n* = 89) of all recommendations were rejected by caregivers and rejection rates of caregivers differed by types of recommendations. Specifically, caregivers’ rejection rate concerning social integration (71.7%) were highest (i.e., caregiver supporting groups). In contrast, caregivers’ rejection rates on recommendations concerning mental health (3.6%), physical health (2.5%), and social, legal, and financial affairs (0%) of caregivers` and PwD were low. Types of caregivers’ rejections were mainly related to personal factors of caregivers (*n* = 66), service factors (*n* = 6), relational factors (*n* = 1), and others (*n* = 17). Referring to caregivers’ rejections due to personal factors, caregivers indicated that the recommendation is “undesired” (*n* = 43) and “unnecessary” (*n* = 43) as well as that they have “no time” (*n* = 12) or already “medical treatment” (*n* = 1). With regard to caregivers’ rejections due to service factors, caregivers stated that service was not available (*n* = 6). With reference to caregivers’ rejections due to relational factors, only one caregiver declared that the recommended support service was not desired by the PwD (*n* = 1). Other factors of caregivers’ rejections included factors that are not included in the adapted and extended HBM model for family dementia caregivers in the present study (based on [13, 14] (for example caregivers refused to talk the factors). The distribution of number, proportion, and types of caregivers’ rejection are shown in Table 2.

Referring to socio-demographic and clinical characteristics of caregivers and PwD, the results of our bivariate analyses showed that a higher number of caregivers’ rejections was significantly associated a higher functional status of PwD (*r* = 0.092, *p* = 0.021). The characteristics of caregivers and PwD as well as bivariate associations between these characteristics and the respective number of caregivers’ rejections are shown in Table 3.

The results of the multivariate analyses confirmed these findings. Specifically, the variable functional status of PwD had a statistically significant effect, with a coefficient of *b* = 0.136 (*p* = 0.011, *CI*_*95−*_ = 0.031, *CI*_*95+*_ = 0.241) (see Table 4). This means that for each one-unit increase in in functional status of PwD, the expected log count of the number of caregivers’ rejection increases by 0.136.

## Discussion

While previous research indicates that rejections of support services mainly results from a lack of acknowledgement of caregivers’ unmet needs and associated recommended tailored support [15, 16], the present study analyzed caregivers’ rejection on tailored support services based on a standardized, computerized unmet needs assessment by dementia-specific qualified nurses. Our results revealed that caregivers’ rejection rate of support services differed by types of recommendations. Caregivers’ rejection rate concerning social integration is high (i.e. joining a caregiver supporting group). Types of caregivers’ rejections were mainly related to personal views of caregivers (i.e., undesired, unnecessary, no time) and service factors (i.e., not available service). Specifically, our results showed that the number of caregivers’ rejections was associated with a higher functional status of the PwD. Thus, our results provide new information on determinants of family dementia caregivers’ rejection of support services, with a particular focus on the number, proportion and types as well as socio-demographic and clinical characteristics.

First, our findings underline the meaning of a comprehensive assessment by dementia-specific qualified nurses including a full range of caregivers’ support services as well as the consideration of caregivers’ socio-demographic and clinical characteristics. In line with previous research [14], our results indicate that dementia-specific qualified nurses should assist family dementia caregivers to access supports and services tailored to their needs in order to increase caregivers’ use of support services.

Second, by following this call for research, the present study implemented a comprehensive dementia case management by qualified nurses providing support to family dementia caregivers to access supports and services tailored to their needs. While caregivers’ rejection rates on recommendations concerning mental health, physical health, and social, legal, and financial affairs were low, caregivers’ rejection rate concerning social integration was high (i.e. caregiver supporting groups). Thus, the rejections of family dementia caregivers are mainly linked to the delegation to caregiver supporting groups. These rejections were mainly related to personal factors of caregivers (i.e. undesired, unnecessary, no time) and service factors (i.e. not available service). Thus, our study emphasizes the urgent need to provide easily manageable as well as financeable support programs that caregivers can get easy access to use and benefit from. As a minimum, family dementia support programs and interventions should include the essential domains of social integration, mental and physical health as well as social, legal, and financial affairs [32]. Despite a comprehensive identification of caregivers’ unmet needs, an individualized tailored support strategy based on these unmet needs, and an accurate information and recommendation on support services for family dementia caregivers from qualified nurses in the present study, the majority of family dementia caregivers rejected participation in caregiver supporting groups (71.7%) due to personal and service factors (e.g., undesired, unnecessary, to time, unavailable service). In line with previous research [12], we suggest that caregiver supporting groups should be established in more flexible and private settings (e.g., telephone- and internet-based, small groups with individual coaching). Furthermore, our results revealed that a higher number of caregivers’ rejections is associated with a higher functional status of the PwD. Thus, caregivers visit supporting groups more often when the PwD shows low abilities in activities of daily living. Importantly, this is independent of the status of cognition and depression of the PwD as well as the physical and mental health of the family dementia caregivers. Accordingly, health care researchers and providers should be aware of this underserved target population and should develop innovative, easily accessible, and personal support for this target group.

Finally, in absence of comprehensive primary data concerning the adapted and extended HBM model for family dementia caregivers (based on [13, 14], the present study empirically validated this theoretical model. Our results confirm that family dementia caregivers’ decisions to reject support depend on four factors: service factors, personal factors, experiential factors, and relational factors. Specifically, we found that mainly personal factors (e.g., caregiver gender, time), service factors (e.g., availability), and relational factors (e.g., preferences of PwD) impact the use and rejection of support service.

### Limitations

Our findings must be interpreted considering several limitations. First, the generalizability of our results might be restricted to family dementia caregivers caring for community-dwelling PwD with mainly mild to moderate cognitive impairments. Family dementia caregivers of PwD in later stages might show different rejection rates as well as associated socio-demographic and clinical characteristics and might benefit from the standardized, computerized assessment by dementia-specific qualified nurses and recommended support services in different ways.

Second, the present analyses are based on data of only two measurement points of a (GP)-based, cluster-randomized intervention trial, and thus, causal relationships between caregivers’ rejections and health-impairments could not be investigated. Furthermore, while caregiving for PwD includes diverse and challenging care tasks that are often associated with a broad range of unmet needs and health-related outcomes, we could not assure to detect every specific existing unmet need in every family caregiver. However, we used a caregivers’ unmet needs assessment including a comprehensive range of domains and validated measurements (e.g., HABC-Monitor [24]) focusing on both the caregiving role domains and health-related outcomes. Furthermore, while we did not measure levels of stigmatization and social desirability of caregivers in detail, future studies should use specific measuring instruments for caregivers’ level of stigmatization and social desirability.

Finally, there is a limitation in the comparability of our results to other health care systems and previous studies analyzing samples from different countries. The standardized, computer-based assessment and the dementia-specific qualification of study nurses have been adapted to the specifics of the German health care system. Thus, future research in other countries and with internationally agreed upon and wider spread measures and recommendations are necessary to compare and validate our findings as well as the HBM model for family dementia caregivers.

## Conclusion

Our results revealed that a standardized, computerized unmet needs assessment by dementia-specific qualified nurses increased the number of caregivers’ agreement from 3% (Lüdecke et al., 2012) to 82% in the present study. Accordingly, in order to provide efficient support services for family dementia caregivers it is necessary to conduct a comprehensive assessment including domains of the caregiving role and health impairments by dementia-specific qualified nurses. A major strength of our study is that it revealed the high number of caregivers’ rejection rate concerning social integration (i.e., caregiver supporting groups) due to personal, service, and relational factors.

Specifically, health care researchers and providers should be aware of the target population with high rejection rates (i.e., higher functional status of PwD) and may develop innovative, easily accessible, and personal support for this target group. Thus, caregivers visit supporting groups more often when the PwD shows low abilities in activities of daily living. Importantly, this is independent of the status of cognition and depression of the PwD as well as the physical and mental health of the family dementia caregivers. While there is an urgent need for easily manageable and available caregivers’ support services concerning social integration (i.e., caregiver supporting groups), future studies must investigate barriers and facilitators for the translation of these targeted interventions in the national health care system.

## Data Availability

The datasets used and/or analysed during the current study are available from the corresponding author on reasonable request.

## Abbreviations

B-ADL: Bayer Activities of Daily Living Scale
BSI-18: Brief Symptom Inventory
DCM: Dementia Care Management
GDS: Geriatric Depression Scale
GP: general practitioner
HBM: Health Behavioral Model
MMSE: Mini-Mental-Status-Test
PWD: people with dementia
SF-12: 12-Item Short Form Survey
